# Equity implications of extended reality technologies for health and procedural anxiety: a systematic review and implementation-focused framework

**DOI:** 10.1093/jamia/ocaf047

**Published:** 2025-03-20

**Authors:** Tom Arthur, Sophie Robinson, Samuel Vine, Lauren Asare, G J Melendez-Torres

**Affiliations:** Department of Public Health and Sports Sciences, Faculty of Health and Life Sciences, University of Exeter, Exeter EX1 2LU, United Kingdom; Department of Public Health and Sports Sciences, Faculty of Health and Life Sciences, University of Exeter, Exeter EX1 2LU, United Kingdom; Department of Public Health and Sports Sciences, Faculty of Health and Life Sciences, University of Exeter, Exeter EX1 2LU, United Kingdom; Department of Public Health and Sports Sciences, Faculty of Health and Life Sciences, University of Exeter, Exeter EX1 2LU, United Kingdom; Department of Public Health and Sports Sciences, Faculty of Health and Life Sciences, University of Exeter, Exeter EX1 2LU, United Kingdom

**Keywords:** health inequities, digital health, anxiety, phobia, realist synthesis

## Abstract

**Objectives:**

Extended reality (XR) applications are gaining support as a method of reducing anxieties about medical treatments and conditions; however, their impacts on health service inequalities remain underresearched. We therefore undertook a synthesis of evidence relating to the equity implications of these types of interventions.

**Materials and Methods:**

Searches of MEDLINE, Embase, APA PsycINFO, and Epistemonikos were conducted in May 2023 to identify reviews of patient-directed XR interventions for health and procedural anxiety. Equity-relevant data were extracted from records (*n *=* *56) that met these criteria, and from individual trials (*n *=* *63) evaluated within 5 priority reviews. Analyses deductively categorized data into salient situation- and technology-related mechanisms, which were then developed into a novel implementation-focused framework.

**Results:**

Analyses highlighted various mechanisms that impact on the availability, accessibility, and/or acceptability of services aiming to reduce patient health and procedural anxieties. On one hand, results showed that XR solutions offer unique opportunities for addressing health inequities, especially those concerning transport, cost, or mobility barriers. At the same time, however, these interventions can accelerate areas of inequity or even engender additional disparities.

**Discussion:**

Our “double jeopardy, common impact” framework outlines unique pathways through which XR could help address health disparities, but also accelerate or even generate inequity across different systems, communities, and individuals. This framework can be used to guide prospective interventions and assessments.

**Conclusion:**

Despite growing positive assertions about XR’s capabilities for managing patient anxieties, we emphasize the need for taking a cautious, inclusive approach to implementation in future programs.

## Background

Digital technologies offer wide opportunities for improving patient outcomes and quality of care. A notable example is extended reality (XR), which is gaining popularity as a patient-facing method of reducing anxieties about medical treatments and conditions.^1^ However, as digital technologies like XR are beginning to be adopted at scale, there is a need to assess their impact on new and existing sources of health inequity. Here, inequity refers to unjust inequalities relating to various social determinants (eg, gender, socioeconomic status, ethnicity), which primarily arise from structural issues and systems of oppression.^2^ Indeed, while empirical assessments of XR-based anxiety interventions have reflected positive outcomes, on average, reported effects are heterogeneous^3^ and systematic reviews imply that these programs may not be equally appropriate for diverse users and services (eg, references^4^^,^^5^). Nevertheless, extant trials predominantly center on efficacy data^1^ and issues that are individual-facing, thereby overlooking the broader factors that could help address unequal patient outcomes and promote equitable implementation of these technologies in the future.

Recent conceptual models, such as Richardson’s digital equity framework^6^ and the Metaverse equitable rehabilitation therapy framework,^7^ have advanced understanding about the complex underlying structures and conditions through which digital services generate health disparities. These accounts outline various technology-related determinants of equitable health and service provision, which span across individual, interpersonal, community, and societal domains. Yet, from an implementation perspective, there is a lack of evidence detailing how different intervention elements and equity mechanisms combine to affect inequitable patient outcomes. This is crucial for developing programs and initiatives that effectively address health disparities.^8^ Hence, there is a need to translate evaluative data and etiological accounts of digital health inequities into prospective models that can shape ongoing efforts to implement and develop equity-generating technologies.

The Tanahashi model^9^ provides an established framework for assessing health service implementation from an equity and effectiveness perspective. The model deconstructs the quality and utilization of interventions into facets that span individual and structural factors. Specifically, preimplementation assessments can center on the degree to which services are made available to the target population by a health system (*availability*), the capacity for patient groups to reach and use these services (*accessibility*), and whether individuals are willing to use the services over time (*acceptability*). By assessing these distinct facets, an array of implementation-focused pathways can be identified and used to inform future policy and service improvements.

## Objective

The present review provides a synthesis of evidence relating to the equity implications of patient-directed XR interventions for health and procedural anxiety. Here, we focused on equity-relevant data pertaining to the management of both general health anxieties (ie, interventions seeking to reduce distress about the implications of a medical condition^10^) and procedural anxieties (ie, interventions seeking to reduce acute and excessive fear of a specific medical or surgical procedure^11^). We used a realist orientation, informed by the Tanahashi model,^9^ to better understand how and why these interventions can support or worsen opportunities for patients with diverse backgrounds and in diverse health system contexts. Through this approach, various equity-relevant *mechanisms* were identified, reflecting the underlying physical, social, or psychological processes assumed to explain individual-, group-, and/or system-level differences in service outcomes. These equity-relevant mechanisms were then developed into a novel, implementation-focused framework that can guide prospective assessments and implementation. Contrary to existing work in the field (eg, the models and theories reviewed by Gustafson et al.^12^) our framework centers on potential equity- and inequity-generating pathways that are specifically relevant to XR technologies. By taking an implementation-focused approach, the framework can guide how these digital technologies are delivered, assessed, and procured within future programs, both within the specific context of managing patient anxieties, and potentially in broader digital health domains.

In short, the review aimed to address the following research question: What are the equity implications of patient-directed XR interventions for health and procedural anxiety, in relation to the availability, accessibility, and acceptability of services?

## Materials and methods

We undertook an equity synthesis broadly underpinned by a realist orientation; that is, a methodology which seeks to understand how, why, and under what conditions interventions work by analyzing their underlying mechanisms, contextual factors, and outcomes.^13^ This analysis formed part of a larger project focusing on the use of XR interventions for managing patient health and procedural anxiety. Here, the precise scope was to systematically examine evidence focusing on the equity implications of these types of interventions, and a bespoke review protocol was preregistered on the Open Science Framework (OSF; see https://osf.io/nhzf8/) to address these objectives. Specifically, the present analysis synthesized review- and trial-level components to identify mechanisms by which XR-based anxiety interventions could provide or worsen opportunities for everyone to attain the highest level of health and wellbeing possible (across diverse backgrounds and health system contexts). Therefore, while our resulting findings and framework may be applicable to wider services and technologies, our methods were originally designed to address this particular clinical “use case” and type of intervention.

Throughout our analyses, we employed the PROGRESS-Plus heuristic to identify equity-relevant characteristics (ie, dimensions of individuals and populations that “stratify health opportunities and outcomes”^14^). PROGRESS-Plus includes: place of residence; race, ethnicity, culture, or language; occupation; gender or sex; religion; education; socioeconomic position; and social capital as key characteristics, and highlights as well the importance of personal characteristics associated with discrimination, features of relationships that might give rise to unequal health opportunities, and time-dependent relationships.^15^ While our broad characterization of intervention mechanisms and context features resembles that of conventional realist approaches (ie, program “theory of change” constructs^13^) the incorporation of the PROGRESS-Plus heuristic and Tanahashi model^9^ as midlevel frameworks scaffolded inductive analyses to be aligned with predefined, equity-specific themes. This approach aligns with more contemporary realist perspectives (eg, “framework analysis” approaches outlined by Nielsen and Lemire^16^) and lent structure to the analysis of why, for whom, and in what circumstances an XR intervention “works.” As a result, specific context-mechanism-outcome relationships could be configured in a manner that directly informs prospective technology implementation and policy developments.

### Search methods and selection criteria

We searched for (1) systematic reviews (2) that examined at least 1 XR-based intervention; (3) focused on patient-directed outcomes for health and procedural anxiety; and (4) were published as full texts from 2013 onward (see accompanying report^1^). Extended reality was defined as any technology that alters a person’s sensory experience by adding immersive, 3D digital assets to a physical or simulated environment (including virtual reality, mixed reality, and augmented reality). To maximize applicability, our screening procedures included reviews that placed sufficient focus on these types of interventions (where XR-based data were analyzed as a discrete category or subgroup), as long as the technology was directly used by patients. However, nonimmersive forms of simulation were excluded (eg, mobile or tablet applications).

From a study design perspective, reviews were eligible if they performed adequate searches of an electronic database, with structured search query and eligibility criteria evidenced. To broaden generalizability, there were no restrictions placed on the language or region in which records were produced, nor on the population or study type reviewed. Furthermore, from an outcome perspective, we included studies of *both* general health anxieties and procedure-specific anxieties. Nonetheless, to maintain consistency, evidence based solely on nonspecific measures of anxiety (eg, fear scales, stress questionnaires) or physiological indicators (eg, heart rate) were excluded.

Searches were conducted on May 30, 2023 for relevant evidence in MEDLINE, Embase, APA PsycINFO (Ovid platform), and Epistemonikos. Appropriate terms were devised for the intervention, outcome, and study design criteria outlined above, and are fully specified at https://osf.io/nhzf8/. Retrieved records were screened independently and in duplicate by 2 reviewers, both at title and abstract stage and at full-text stage. Instances of disagreement between reviewers were resolved via recourse to a third reviewer. Reasons for exclusion at full-text stage were recorded and are also available on the project OSF page (at https://osf.io/nhzf8/).

### Data extraction

#### Review-level analysis

Equity-relevant data were extracted from all of the systematic reviews identified from the literature searches. Using free-text coding in Nvivo, we specifically extracted statements that captured how some aspect of a relevant intervention, such as its design, implementation, or effectiveness, relates to an equity-relevant characteristic (ie, any of those listed in the PROGRESS-Plus heuristic, described above).

#### Within-review analysis

Next, we identified 5 of the largest, best and most recent reviews to initiate synthesis of trial-level data. This prioritization process was conducted by 2 reviewers, independently and in duplicate, who considered the number of studies evaluated and the diversity of analyses/outcomes performed within each included article. Reviews by Addab et al.,^17^ Koo et al.,^18^ Kılıç et al.,^19^ Ahmad et al.,^20^ and Wang et al.^21^ were chosen on this basis. Full-text records were subsequently retrieved in cases where the primary studies were relevant to health or procedural anxiety. Statements relating to equity-relevant characteristics were then extracted, as above, using free-text coding in Nvivo.

### Equity synthesis

Initially, extracted statements were deductively categorized using several axes: (1) review-level or within-review extraction; (2) equity-relevant characteristics (defined by PROGRESS-Plus); (3) whether statements relate to availability, accessibility, or acceptability of relevant XR interventions (as defined by the Tanahashi model^9^) and (4) whether statements relate to availability, accessibility, or acceptability of the health or procedural contexts linked to XR interventions.

Following initial categorization, statements were placed into grids defined by different axes of categorization. Consistent with our realist orientation and equity-focused frameworks, we specifically classified mechanisms by contextual factors, relating to: (1) which “side of the equation” they reflected and (2) which kinds of equity-relevant characteristics were linked to mechanism. To classify mechanisms by “side of the equation,” we described those related to the *target situation*, that is, the health condition or service delivery problem for which a digital solution is sought, and those related to the *focal technology*, or the proposed digital solution. We then organized the mechanisms into outcomes, based on whether the equity-relevant characteristics related to availability, or health system-related characteristics; accessibility, or group and population-level characteristics; and acceptability, or individual-level and situational characteristics. Since most XR-based patient anxiety interventions have not yet been implemented within large-scale services, characteristics relating to contact coverage (ie, who has *reached* the service at their time of requirement) and effectiveness coverage (ie, who has actually *used* the service) from Tanahashi’s original model^9^ were not examined.

Once formed, the categorized grids were used to develop a conceptual framework, characterizing the potential equity- and inequity-generating impacts of XR interventions for health and procedural anxiety. Analysis was inductive and consensual in nature: specifically, a hierarchical coding strategy was used to cluster similar statements and generate higher order findings relating to equity mechanisms and their associated contextual factors. Here, we also considered whether findings vary by the labels applied to included systematic reviews, to identify where and why, for example, interventions may be inequity-generating for one type of intervention but not for another. All of the concepts outlined in our ensuing framework emerged from these inductive analytical processes, and the interpretation of categorized equity-relevant data by our review team.

### Patient and public involvement

Before undertaking this review, we led a public involvement workshop with clinicians and patients with lived experience of health and/or procedural anxieties. This workshop contributed to the design of our equity syntheses, through highlighting possible disparities in health services and XR interventions. As these involvements sit within the scope of planning and advisory activities (according to the UK National Institute for Health and Care Research), ethical approval was not required. Specifically, the stakeholders provided insight on potential sociocultural barriers (eg, language issues, boundaries imposed by race and religion), economic constraints, accessibility issues (eg, for patients with complex medical conditions), and health care-related attitudes/behaviors (that may differ between individuals or communities) that warrant consideration.

## Results

### Search results and included studies

**Figure 1.**
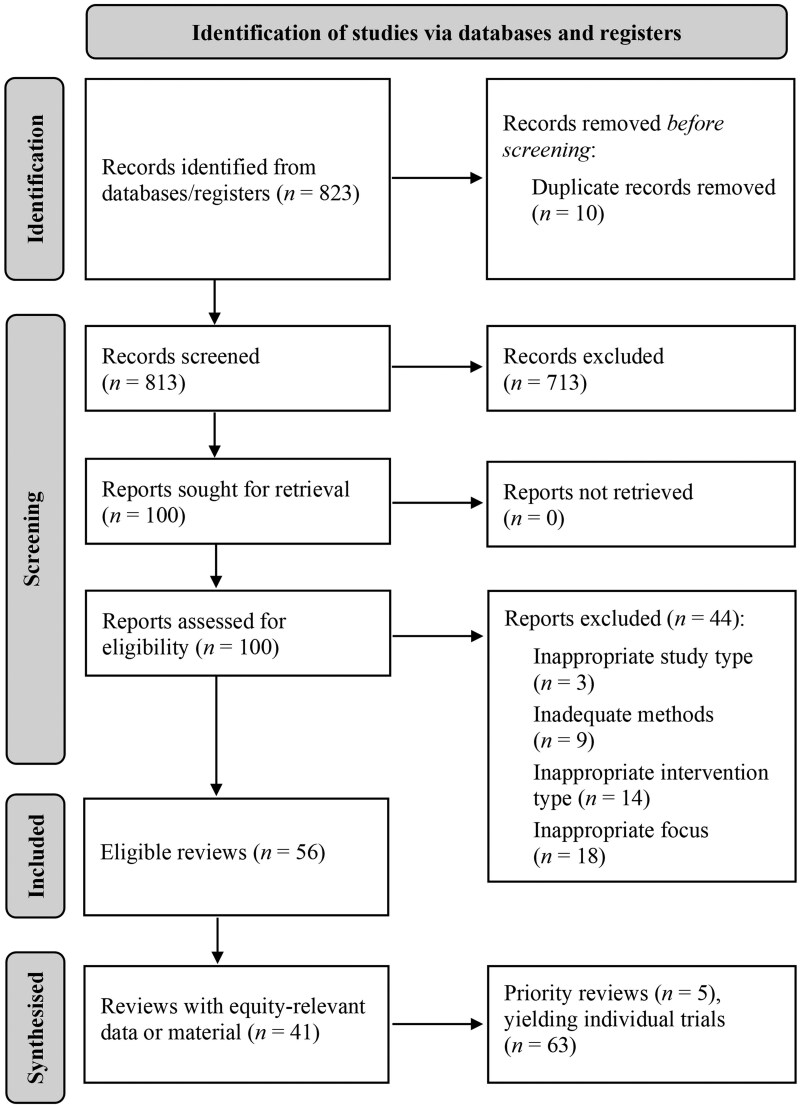
PRISMA flow diagram of retrieved, screened, and included review articles.

A PRISMA flowchart of the search process is presented in Figure 1, and a full list of included studies is presented in Appendix S1. In summary, 56 reviews were retrieved from our searches, all of which were published between 2018 and 2022. Most of these reviews included meta-analyses (*n *=* *35), though scoping reviews (*n *=* *5) and narrative syntheses (*n *=* *16) were also included. A further 63 trials were then identified from the 5 priority reviews that met inclusion criteria. While these trials utilized wide-ranging methods, in diverse clinical scenarios, they all examined patient-directed XR technologies within the target context (as a method for reducing health or procedural anxiety).

Of the eligible records, 41 reviews and 59 trials reported equity-relevant data that could be synthesized in our analyses. While most of these articles performed statistical assessments of equity-related data, such analyses were typically confined to simple comparisons of demographic descriptors (eg, to check that randomized groups comprised of similar ages, gender, and ethnicity profiles). Only 28/100 articles (6 reviews, 22 trials) directly examined or adjusted for the effects of equity-relevant variables on intervention outcomes, and even fewer (13/100 articles) presented direct subgroup comparisons or covariance analyses of anxiety-specific data. Moreover, some disadvantaged groups were excluded from studies altogether, such as patients with chronic medical conditions and/or disabilities (eg, mental health issues, stroke history, epilepsy, or physical frailties).

### Equity-relevant mechanisms

#### Mechanisms pertaining to the target situation

Throughout the included literature, numerous equity-relevant mechanisms linked to the target situation—often associated with rationales for studying or evaluating XR technologies—were offered (Table 1). *Availability mechanisms* typically related to the capacity of health services to feasibly provide effective interventions, which may be limited by financial resources, staffing/facilities, policy priorities (eg, needs for efficiency gains), or workflow constraints. Notably, disparities in the prevalence of health conditions and patient anxieties (eg, between low- and high-income regions) may further impact on these service capacities.^19^^,^^39^^,^^60^^,^^61^  *Accessibility mechanisms* related to dispossessed groups’ inability to take up health services, due to factors like mobility and/or transport issues,^32^^,^^52^ co-occurring health conditions,^22^ and time demands.^54^^,^^55^ Again, these barriers can be further impacted by imbalances in health-care needs between groups (eg, due to differences in the prevalence of health conditions; see Table 1). *Acceptability mechanisms* related to individual or situational preferences. Indeed, acceptance and adherence may be influenced by patient characteristics (eg, relating to age, education, socioeconomic position, language abilities^31^^,^^46–48^) as well as their experiences during care (eg, any adverse effects or inappropriate treatment designs^23^^,^^41^^,^^49^^,^^56^^,^^57^^,^^59^).

**Table 1.** Summary of findings of XR-based health and procedural anxiety interventions: possible inequity-generating impacts (mechanisms) pertaining to the target situation (context).

Equity-relevant characteristics (contextual factors)	Availability outcomes	Accessibility outcomes	Acceptability outcomes
***Health service factors***	Costs and budgets impact on the ability to offer timely and effective treatments^22–27^	Treatment demands are unequally distributed between low- and high-income areas^28^^,^^29^	Patients are not always comfortable with the most practical, safe, or efficient treatment methods^30^
	Demands/priorities are unequal between low- and high-income regions^25^	Effective preoperative and screening interventions can require additional time and visits from patients^22^^,^^31^	
	The ability to offer effective care programs is reduced for providers with limited staffing or support capacities^22^^,^^32–36^		
	Interventions are not always available or realistic within clinical settings^27^^,^^37^		
***Age***	Effective treatment methods can be less readily available for young children^38^	The need to undergo health treatments can be greatest in early childhood^38–40^ and later adulthood^41–44^	Negative health-care experiences are more commonly faced by children^17^^,^^35^^,^^45^
			Children show poorer compliance/adherence to interventions^46^^,^^47^
***Gender***	No relevant data presented in the included reviews	The prevalence of health conditions (and treatment needs) can be unequal between different genders^42^^,^^44^^,^^48^	No relevant data presented in the included reviews
***Disabilities and health conditions***	No relevant data presented in the included reviews	Needs to undergo treatment are greater for those with chronic or co-occurring health conditions^49^	Some patients (eg, autistic people) have added needs/challenges cooperating with health staff or treatments^37^
		Interventions can exclude patient groups; for example, those with limited mobility^32^ or weakened immunity^22^	
***Race/Ethnicity***	No relevant data presented in the included reviews	The prevalence of health conditions (and thus, the need to undergo treatment) is greater in some ethnic groups^28^	No relevant data presented in the included reviews
***Socioeconomic position and cultural factors***	No relevant data presented in the included reviews	Health conditions (and thus, the need to undergo treatment) can be more common in certain populations,^50^ for example, when comparing between low- and high-income groups^28^^,^^29^	Care is not always tailored for individual language preferences or communication abilities^37^^,^^51^
		Service access is limited for patients with transportation or mobility issues^52^	Negative care experiences can be common in patients with lower education levels^31^^,^^48^^,^^50^^,^^53^
		Uptake of care is lower for those with limited time availability^54^^,^^55^	
***Adverse effects***	Side effects place added demands on service providers^23^^,^^56–58^	Side effects can have inequitable impacts on certain patient groups^49^^,^^53^	Adverse effects and unpleasant experiences are common for many treatments^23^^,^^41^^,^^49^^,^^56–59^

Abbreviation: XR, extended reality.

#### Mechanisms pertaining to the focal technology

Various mechanisms linked to the focal technology were adduced from the evidence. These included both equity- and inequity-generating pathways to impact (see Tables 2 and 3). For example, *availability mechanisms* related to health system capacity to invest in, staff, or otherwise maintain XR technologies; or the ability to prioritize their introduction into clinical practice. The reviewed evidence mostly highlighted benefits in this area, relating to the relative inexpensiveness, feasibility, and flexibility of the technology for adoption (see Table 3). Moreover, researchers emphasized the potential for XR-based patient anxiety therapies to facilitate treatments that are safer, quicker, and more cost-effective for providers (eg, by reducing procedure times, side effects, and the need for further analgesic intervention^23^^,^^32^^,^^35^^,^^38^^,^^51^^,^^59^^,^^71^^,^^76^^,^^79^^,^^80^^,^^86^^,^^89^^,^^99^^,^^102^). Nonetheless, it was noted that there can be large upfront costs associated with the technology, and that its implementation can require private space, robust networking, dedicated infection control measures, and assigned time to set up and deliver (see Table 2). Although standalone XR applications are said to require minimal staffing support and/or service resources,^32^^,^^41^^,^^47^^,^^71^^,^^76^ it is noted that many interventions are still carried out within a hospital setting and under specialist staff supervision.^34^

**Table 2.** Summary of findings of XR-based health and procedural anxiety interventions: possible inequity-generating impacts (mechanisms) pertaining to the focal technology (context).

Equity-relevant characteristics (contextual factors)	Availability outcomes	Accessibility outcomes	Acceptability outcomes
***Health system factors***	Can come with large upfront costs^4^^,^^24^^,^^26^^,^^27^^,^^62–66^	The costs and resources required to engage with XR programs could exclude some patient groups,^26^^,^^39^^,^^63^^,^^64^ unless absorbed by relevant provider	No relevant data presented in the included reviews
	Private space^67^ and robust Wi-Fi networks or tethering^39^^,^^67^^,^^68^ may be needed		
	Can require specialist staffing,^4^^,^^26^^,^^35^^,^^49^^,^^53^^,^^64^^,^^65^^,^^68–70^ but still demands relatively little of their time^41^^,^^47^^,^^71^		
	Needs time to set up and/or deliver^27^^,^^35^^,^^68^^,^^72^^,^^73^		
	Can interfere with clinical workflows^24^^,^^35^^,^^62^^,^^73^ and present infection control risks^4^^,^^35^^,^^49^^,^^64^^,^^74^^,^^75^		
***Age***	Safe and appropriate applications are not yet available for all age groups^4^^,^^69^	Eligibility criteria for XR interventions may exclude older patients with frailties^74^	Tools can be unsuitable for young children^4^^,^^25^^,^^28^^,^^32^^,^^64–67^^,^^73^^,^^76–78^
			Older adults may show poor acceptance and/or usability^5^^,^^21^^,^^24^^,^^30^^,^^51^^,^^62^^,^^64^^,^^74^
			Older children and adults may be less engaged^18^^,^^23^^,^^59^^,^^79–85^
***Gender***	No relevant data presented in the included reviews	No relevant data presented in the included reviews	The perception and utilization of XR tools may vary between genders^5^^,^^18^^,^^27^^,^^86^
			Apps do not always tailor content to gender differences^68^^,^^87^^,^^88^
***Disabilities and health conditions***	No relevant data presented in the included reviews	Some patients may be ineligible for XR use (eg, due to history of epilepsy or seizures)^24^^,^^27^^,^^74^^,^^89^^,^^90^	Variability may relate to prior trauma^55^^,^^91^ or psychiatric conditions^55^^,^^92^
		May not be inclusive for all patients,^93^^,^^94^ such as those with cognitive delays,^17^ alopecia,^95^ injuries,^27^^,^^40^^,^^64^ or glasses wearers^39^	
***Sociocultural factors***	No relevant data presented in the included reviews	Can exclude those with limited digital skills and experience^30^^,^^62^^,^^72^^,^^74^^,^^96^	Can place added engagement demands/inconvenience onto patients^49^^,^^72^^,^^73^^,^^97^^,^^98^
		Time constraints may exclude some patients from taking up these interventions^97^	Variability may relate to social factors^5^^,^^33^^,^^59^ and education levels^33^^,^^48^^,^^55^
			Content is not usually tailored for different cultures or ethnicities^4^^,^^27^^,^^40^^,^^88^
			Apps may be rudimentary for patients who are “digital natives”^62^
***Adverse effects***	No relevant data presented in the included reviews	Cybersickness may be more common in certain groups^52^	Cybersickness and minor discomfort can be experienced^26^^,^^30^^,^^32^^,^^35^^,^^40^^,^^50^^,^^52^^,^^64^^,^^72^^,^^74^^,^^75^^,^^77^^,^^79^^,^^84^^,^^88^^,^^97^^,^^99–101^

Abbreviation: XR, extended reality.

**Table 3.** Summary of findings of XR-based health and procedural anxiety interventions: possible equity-generating impacts (mechanisms) pertaining to the focal technology (context).

Equity-relevant characteristics (contextual factors)	Availability outcomes	Accessibility outcomes	Acceptability outcomes
***Health system factors***	Inexpensive compared to alternative interventions^19^^,^^22^^,^^23^^,^^25^^,^^33^^,^^35^^,^^36^^,^^39^^,^^41^^,^^49^^,^^51^^,^^56–58^^,^^70^^,^^71^^,^^74–76^^,^^80^^,^^86^^,^^89^^,^^95^^,^^96^^,^^102–108^ and can facilitate cost-effective care pathways^25^^,^^30^^,^^35^	XR is becoming more reasonably priced^33^^,^^35^^,^^36^^,^^86^^,^^103^^,^^105^^,^^106^ and readily obtainable^35^^,^^69^^,^^86^ for the general public	Location and timing of delivery can be flexible^24^^,^^36^^,^^37^^,^^54^^,^^55^
	Readily obtainable for service providers^33^^,^^39^^,^^70^^,^^75^^,^^86^^,^^103^		Willingness to uptake and adhere to interventions appears high in low-income nations^99^
	Does not need to take place in busy clinical settings^32^^,^^33^^,^^65^^,^^76^		
	Feasible^46^^,^^49^^,^^80^^,^^108^ and scalable,^32^^,^^70^ but see counter arguments^65^^,^^74^		
	Reduces treatment time^23^^,^^32^^,^^38^^,^^51^^,^^59^^,^^76^^,^^79^^,^^80^^,^^89^		
	Allows safe procedures to be performed^30^ without added interventions or constraints^38^^,^^71^^,^^79^^,^^100^^,^^102^		
***Age***	No relevant data presented in the included reviews	No relevant data presented in the included reviews	Younger users may be more comfortable and engaged^18^^,^^23^^,^^35^^,^^37^^,^^79–84^
***Disabilities and health conditions***	No relevant data presented in the included reviews	Greater accessibility of care for users with mobility issues,^32^ autism,^37^ infection risks^4^^,^^42^^,^^94^	Can be tailored for diverse phobias^75^ and needs^52^^,^^94^
***Sociocultural factors***	No relevant data presented in the included reviews	Can be accessed remotely, thereby removing mobility and transport barriers^24^^,^^32^^,^^37^^,^^42^^,^^47^^,^^52^	Applications can be adapted for different language abilities or preferences^48^^,^^51^
		Can convey challenging concepts^31^^,^^51^ to users with low health literacy^24^^,^^51^ or knowledge^47^^,^^85^	Sessions can be quick and easy to complete^41^^,^^69^^,^^76^
		Can be accessible for those with minimal prior XR experience^54^^,^^85^	
***Adverse effects***	Adverse effects are less serious and/or consequential than in other interventions^23^^,^^35^^,^^76^	No relevant data presented in the included reviews	Relatively few or minimal adverse effects are generally experienced^20^^,^^21^^,^^26^^,^^27^^,^^32^^,^^35^^,^^36^^,^^39^^,^^41^^,^^44^^,^^48^^,^^50–53^^,^^56^^,^^57^^,^^59^^,^^62^^,^^65^^,^^71^^,^^73^^,^^75^^,^^77–81^^,^^85–87^^,^^90^^,^^95^^,^^97–101^^,^^105^^,^^108–112^

Abbreviation: XR, extended reality.

*Accessibility mechanisms* related to common determinants of health equity, such as digital literacy and resources necessary to take up new interventions. Additional costs and time demands could be placed on patients that act as a barrier for uptake, while certain groups may be excluded by XR-specific eligibility criteria (eg, for those with a history or seizures or frailty-related issues^4^^,^^24^^,^^27^^,^^74^^,^^90^) and prerequisite skills or experience with the technology (eg, for some individuals that are elderly or from specific socioeconomic backgrounds^5^^,^^24^^,^^30^^,^^62^^,^^74^). The ability to use XR remotely does, however, offer benefits for patients with mobility or transport issues, in addition to those who have specific clinical risks (eg, patients who are immunosuppressed^22^) and/or needs (eg, autistic people who have sensory and sociocommunicative challenges^37^).

Finally, *acceptability mechanisms* related to adverse effects, such as cybersickness, that might generate inequity in implementation settings where digital technologies are the only supported option. The fact that some patient groups may be more susceptible to these negative experiences was highlighted (eg, Wu et al.^52^) though adverse effects were generally deemed rare and inconsequential (compared to other treatment methods). Similarly, while acceptance was seen to be lower in certain patient populations (eg, elderly adults, older children, women, patients with co-occurring conditions; see Table 2), contradictory findings emerged in the reviewed literature, and high levels of patient engagement, satisfaction, and adherence were generally reported. Moreover, specific equity-generating mechanisms were identified from the unique ability to tailor XR applications for diverse language backgrounds and individual preferences (eg, by using standalone devices that can be flexibly deployed at a time and location of choosing^24^^,^^36^^,^^37^^,^^54^^,^^55^).

### The double jeopardy, common impact framework

**Figure 2.**
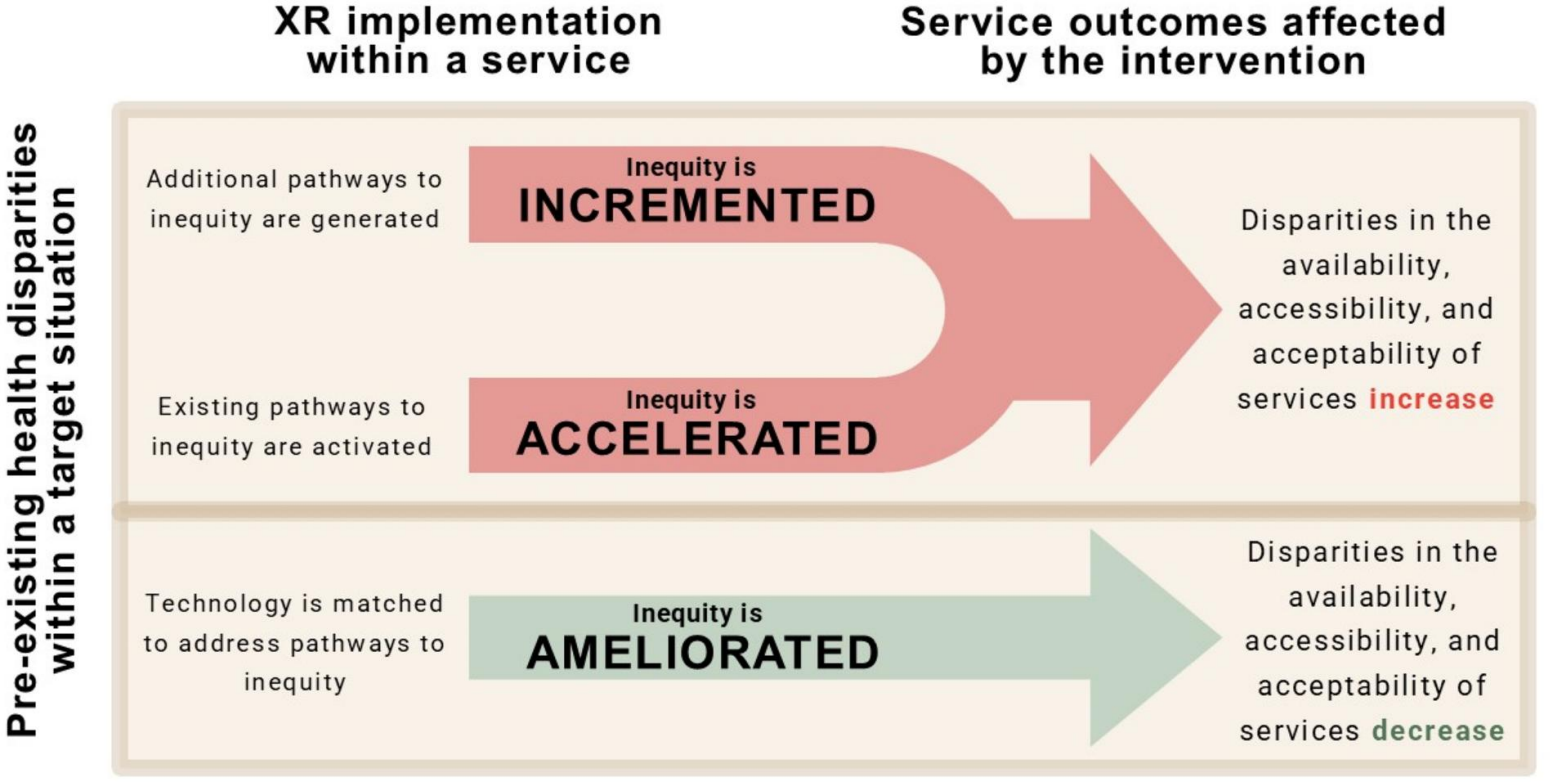
Schematic overview of the double jeopardy, common impact framework.

Following the identification and classification of mechanisms (outlined above), results were developed into a conceptual framework that characterized potential equity- and inequity-generating impacts of XR interventions for health and procedural anxiety. Here, our review team inductively examined findings that spanned across different mechanisms, outcomes, and clinical contexts, noting patterns of discrepancy and cohesion that emerged (eg, across both “sides of the equation,” as detailed in “Materials and Methods”) and interpreting their impacts from an implementation-focused perspective. This process identified 3 key pathways based on combinations of mechanisms arising from the link between a target situation and a focal technology: 2 double jeopardy pathways and 1 common impact pathway (illustrated in Figure 2).

#### Double jeopardy: accelerating inequity

Firstly, a focal technology could activate inequity-generating mechanisms in the same domain as in the target situation. For example, in situations where accessibility-based mechanisms are key due to socioeconomic disparities in access to care, introducing an XR-based anxiety intervention that relies heavily on advanced digital literacy, or on extensive use of mobile data, will accelerate accessibility-related inequities for already disadvantaged groups. Alternatively, at a systemic level, the implementation of digital technologies that require specialist staffing and/or dedicated user environments (eg, private spaces, robust networking) may be particularly challenging for services that are overstretched, thereby exacerbating intergroup health inequities.

#### Double jeopardy: incrementing inequity

In another instance, a focal technology could generate additional pathways to inequity. For example, in a situation where availability-based mechanisms drive uptake of focal technologies, the implementation of a technology that generates inequities by accessibility will worsen social gradients in health services and outcomes during XR-based anxiety interventions. Unequal susceptibility to cybersickness and XR-related discomfort may further introduce disparities in terms of acceptance and accessibility. Health systems will also need to consider the opportunity cost that is generated by investing in technologies that do not directly address the equity-relevant mechanisms in the target situation.

#### Common impact: matching inequity with equity

Finally, focal technologies can be designed to directly address inequity-generating mechanisms in the target situation. Here, optimal fit between the focal technology and the target situation can ameliorate inequity. For example, by implementing an XR-based anxiety intervention that can be performed in patients’ homes, and at a time of their choosing, it may be possible to reduce common access barriers (eg, relating to transport or mobility issues, time constraints, or infection risks). Similarly, XR content can be designed and adapted based on individual preferences (eg, different language abilities, education levels, sensory needs) to address disparities in acceptance and maximize uptake across diverse user populations.

## Discussion

The present research provides an equity-focused syntheses of evidence relating to patient-directed XR interventions for health and procedural anxiety. Here, salient situation- and technology-based mechanisms were identified that could impact on the broad availability, accessibility, and acceptability of service coverage. Our “double jeopardy, common impact” framework models how these mechanisms combine to accelerate, increment, or improve equity outcomes. On one hand, XR solutions offer unique opportunities for addressing inequity-generating mechanisms in the target situation, especially those concerning transport, cost, or mobility barriers. However, these interventions can also accelerate areas of inequity that link to health outcomes, or even engender additional disparities at systemic, group, or individual levels. Taken together, our results indicate that XR interventions are unlikely to be a panacea, and emphasize the need for taking a cautious, inclusive approach to implementation in future services.

The proposed framework adopts an *implementation-focused* approach that can guide future research and practice. Firstly, it captures key concepts developed in recent technology-based models, such as the individual, interpersonal, community, and societal determinants of health identified in Richardson’s digital equity framework,^6^ and respective subdomains presented in the Metaverse equitable rehabilitation therapy framework.^7^ Crucially, we have then developed these theoretical concepts into explanatory mechanisms of action,^113^ which detail how diverse elements of XR-based patient anxiety interventions combine to affect health equity outcomes. Thus, instead of concentrating on specific system features or user experiences, the proposed framework describes how XR technologies interact with contextual factors and individual circumstances to improve or worsen patient opportunities (in a manner that is applicable to numerous digital interventions and use cases). As a result, it can implicate how XR systems are designed for future applications, while also informing how they are delivered, assessed, and/or procured within wide-ranging clinical programs.

Notably, our equity synthesis develops generalized constructs from health implementation models, such as the CFIR^114^ and PARIHS^115^ frameworks, into intervention-specific themes and pathways that could guide future practice. We highlight that there are clear opportunities for reducing health disparities via targeted XR solutions; however, these potential benefits are by no means guaranteed in patient anxiety interventions and there are existing or additional sources of inequity that may limit such endeavors. For instance, while home-based XR interventions may lower costs associated with attending and providing services, lower digital literacy exhibited within some community groups (eg, those with poorer socioeconomic or education backgrounds^116^) could limit positive, equitable outcomes from being experienced. Similarly, opportunities for improving inclusivity and accessibility of care (eg, for those with specific mobility issues or sensory needs) could be negated by heavy and unwieldly hardware systems that can produce adverse effects (eg, cybersickness, physical discomfort). Evaluations of new technologies should therefore consider how design relates to availability, accessibility, and acceptability factors through inclusive technology development and implementation.

Nonetheless, XR systems represent promising and fast-evolving digital solutions that are being increasingly applied within health-care settings. Applications of these technologies are no longer confined to specialized clinical contexts, but extend into diverse areas of care navigation and service provision.^117^ Despite the need for further research, our framework presents key, implementation-focused pathways that can be generalized across various use cases to better address disparities in the availability, accessibility, and acceptability of care. In the specific context of health anxieties, our results highlight precise mechanisms for potentially enhancing patient inclusion and equity outcomes. Indeed, while empirical data indicate that these types of interventions are already generally effective,^1^^,^^3^ we have identified focal, equity-relevant themes that impact on their uptake, inclusivity, and overall efficacy. It is essential that these issues are proactively considered within future policy and practice, to ensure that innovative XR solutions offer maximum benefit to all service providers and patient populations.

The main strengths of this research lie in its rigorous methods, whereby extensive review- and trial-level evidences have been systematically retrieved and synthesized by an interdisciplinary team of health scientists and immersive technology specialists (who were informed by patient and public engagement activities). This comprehensive approach ensured that our “double jeopardy, common impact” framework was underpinned by diverse empirical data and robust analyses. However, from a limitation’s perspective, it should be recognized that the associated underlying data concern a specific use case of XR (ie, patient-directed interventions for the management of health and procedural anxieties). Furthermore, trial-level synthesis was limited to data from only 5 priority reviews, meaning that relevant study findings may have been overlooked. Thus, generalizability to other target situations, study designs, and technology combinations merits future evaluation, alongside a broader need for equity-focused research on novel health technologies.

## Conclusion

Overall, despite the advancing utility of XR within health and procedural anxiety interventions, their capacity for promoting greater availability, accessibility, and acceptability of care for everyone, everywhere, requires further attention. Our “double jeopardy, common impact” framework details how targeted XR solutions offer tangible pathways for addressing health disparities in this domain, but also focal pathways for accelerating inequalities and generating additional disparities in service coverage and/or uptake. This framework provides clear implications for future research and practice, where significant developments are needed to optimize the design and delivery of inclusive XR innovations.

## Supplementary Material

ocaf047_Supplementary_Data

## Data Availability

The data underlying this article are available on the Open Science Framework, at https://osf.io/nhzf8/.
