# Supplementary material for: Equity implications of extended reality technologies for health and procedural anxiety: a systematic review and implementation-focused framework
Source: J Am Med Inform Assoc. 2025 Mar 20;32(5):945–57. doi: 10.1093/jamia/ocaf047 (PMC12012361; doi:10.1093/jamia/ocaf047)
Supplement: ocaf047_Supplementary_Data [file ocaf047_supplementary_data.docx]

**APPENDIX 1: LIST OF INCLUDED STUDIES**

| **Supplementary Table 1.** Review articles included in our analyses. | | |
| --- | --- | --- |
| **Authors** | **Year** | **Article Title** |
| Addab, S., Hamdy, R., Thorstad, K., Le May, S., Tsimicalis, A. | 2022 | Use of virtual reality in managing paediatric procedural pain and anxiety: An integrative literature review***** |
| Ahmad, M., Mohammad, E., Anshasi, H. | 2020 | Virtual reality technology for pain and anxiety management among patients with cancer: a systematic review***** |
| Alqudimat, M., Mesaroli, G., Lalloo, C., Stinson, J., Matava, C. | 2021 | State of the Art: Immersive Technologies for Perioperative Anxiety, Acute, and Chronic Pain Management in Pediatric Patients |
| Baradwan, S., Khadawardi, K., Badghish, E., Alkhamis, W., Dahi, A., Abdallah, K., Kamel, M., Sayd, Z., Mohamed, M., Ali, H., Elhalim, A., Mahmoud, M., Mohamed, A., Mohamed, D. , Shama, A., Hagras, A., Ali, H., Abdelhakim, A., Saleh, M., Badawy, M., Bakry, M. | 2022 | The impact of virtual reality on pain management during normal labor: A systematic review and meta-analysis of randomized controlled trials |
| Bashir, Z., Misquith, C., Shahab, A., Has, P., Bukhari, S. | 2023 | The impact of virtual reality on anxiety and functional capacity in cardiac rehabilitation: a systematic review and meta-analysis |
| Bu, X., Ng, P., Xu, W., Cheng, Q., Chen, P., Cheng, A., Liu, X. | 2022 | The Effectiveness of Virtual Reality-Based Interventions in Rehabilitation Management of Breast Cancer Survivors: Systematic Review and Meta-analysis |
| Chen, J., Xie, Z., Or, C. | 2021 | Effectiveness of immersive virtual reality-supported interventions for patients with disorders or impairments: a systematic review and meta-analysis |
| Chen, Y., Cao, L., Xu, Y., Zhu, M., Guan, B., Ming, W. | 2022 | A systematic review and meta-analysis of randomized controlled trials |
| Chen, Y. J., Wang, C. J., Chen, C. | 2023 | Effects of virtual reality on preoperative anxiety in children: A systematic review and meta‐analysis of randomised controlled trials |
| Comparcini, D., Simonetti, V., Galli, F., Saltarella, I., Altamura, C., Tomietto, M., Desaphy, J., Cicolini, G. | 2023 | Immersive and non-immersive virtual reality for pain and anxiety management in pediatric patients with hematological or solid cancer: A systematic review |
| Cortés-Pérez, I., Zagalaz-Anula, N., Ibancos-Losada, M., Nieto-Escámez, F., Obrero-Gaitán, E., Osuna-Pérez, M. | 2021 | Virtual reality-based therapy reduces the disabling impact of fibromyalgia syndrome in women: Systematic review with meta-analysis of randomized controlled trials |
| Cunningham, A., McPolin, O., Fallis, R., Coyle, C., Best, P., McKenna, G. | 2021 | A systematic review of the use of virtual reality or dental smartphone applications as interventions for management of paediatric dental anxiety |
| Custódio, N., Costa, F., Cademartori, M., da Costa, V., Goettems, M. | 2020 | Effectiveness of virtual reality glasses as a distraction for children during dental care |
| Găină, M., Szalontay, A., Ștefănescu, G., Bălan, G., Ghiciuc, C., Boloș, A., Găină, A., Ștefănescu, C. | 2022 | State-of-the-art review on immersive virtual reality interventions for colonoscopy-induced anxiety and pain |
| Gao, Y., Xu, Y., Liu, N., Fan, L. | 2023 | Effectiveness of virtual reality intervention on reducing the pain, anxiety and fear of needle-related procedures in paediatric patients: A systematic review and meta-analysis |
| Grilo, A., Almeida, B., Rodrigues, C., Gomes, A., Caetano, M. | 2023 | Using virtual reality to prepare patients for radiotherapy: A systematic review of interventional studies with educational sessions |
| Hajesmaeel-Gohari, S., Sarpourian, F., Shafiei, E. | 2021 | Virtual reality applications to assist pregnant women: a scoping review |
| Hao, J., Li, Y., Swanson, R., Chen, Z., Siu, K. | 2023 | Effects of virtual reality on physical, cognitive, and psychological outcomes in cancer rehabilitation: a systematic review and meta-analysis |
| Huang, Q., Lin, J., Han, R., Peng, C., Huang, A. | 2022 | Using virtual reality exposure therapy in pain management: a systematic review and meta-analysis of randomized controlled trials |
| Kılıç, A., Brown, A., Aras, I., Hui, R., Hare, J., Hughes, L. D., McCracken, L. | 2021 | Using Virtual Technology for Fear of Medical Procedures: A Systematic Review of the Effectiveness of Virtual Reality-Based Interventions***** |
| Koo, C. H., Park, J. W., Ryu, J. H., Han, S. H. | 2020 | The effect of virtual reality on preoperative anxiety: A meta-analysis of randomized controlled trials***** |
| Lan, X., Tan, Z., Zhou, T., Huang, Z., Wang, C., Chen, Z., Ma, Y., Kang, T., Gu, Y., Wang, D., Huang, Y. | 2023 | Use of Virtual Reality in Burn Rehabilitation: A Systematic Review and Meta-analysis |
| Leggiero, N., Armstrong, T., Gilbert, M., King, A. | 2020 | Use of virtual reality for symptom management in solid-tumor patients with implications for primary brain tumor research: a systematic review |
| Lluesma-Vidal, M., González, R., García-Garcés, L., Sánchez-López, M., Peyro, L., Ruiz-Zaldibar, C. | 2022 | Effect of virtual reality on pediatric pain and fear during procedures involving needles |
| Lopez-Valverde, N., Fernandez, J., Lopez-Valverde, A., Juan, L., Ramirez, J., Fraile, J., Payo, J., Antona, L., de Sousa, B., Bravo, M. | 2020 | Use of virtual reality for the management of anxiety and pain in dental treatments: Systematic review and meta-analysis |
| Martinez-Bernal, D., Vidovich, C., Keenan, C., Correll, L., Laserna, A., Hasselberg, M., Cross, W., Kolokythas, A. | 2023 | The use of virtual reality to reduce pain and anxiety in surgical procedures of the oral cavity: A scoping review |
| Obrero-Gaitan, E., Cortes-Perez, I., Calet-Fernandez, T., Garcia-Lopez, H., Lopez Ruiz, M., Osuna-Perez, M. | 2022 | Digital and Interactive Health Interventions Minimize the Physical and Psychological Impact of Breast Cancer, Increasing Women's Quality of Life: A Systematic Review and Meta-Analysis |
| Saliba, T., Schmartz, D., Fils, J., Van Der Linden, P. | 2022 | The use of virtual reality in children undergoing vascular access procedures: a systematic review and meta-analysis |
| Scapin, S., Echevarría-Guanilo, M., Junior, P. , Gonçalves, N., Rocha, P., Coimbra, R. | 2018 | Virtual Reality in the treatment of burn patients: A systematic review |
| Simonetti, V., Tomietto, M., Comparcini, D., Vankova, N., Marcelli, S., Cicolini, G. | 2022 | Effectiveness of virtual reality in the management of paediatric anxiety during the peri-operative period: A systematic review and meta-analysis |
| Smith, V., Warty, R., Sursas, J., Payne, O., Nair, A., Krishnan, S., da Silva Costa, F., Wallace, E., Vollenhoven, B. | 2020 | The effectiveness of virtual reality in managing acute pain and anxiety for medical inpatients: systematic review |
| Smith, K., Wang, Y., Colloca, L. | 2022 | Impact of virtual reality technology on pain and anxiety in pediatric burn patients: a systematic review and meta-analysis |
| Tas, F. Q., van Eijk, C. A., Staals, L. M., Legerstee, J. S., Dierckx, B. | 2022 | Virtual reality in pediatrics, effects on pain and anxiety: A systematic review and meta-analysis update |
| Turan Kavradim, S., Yangoz, S., Ozer, Z. | 2023 | Effectiveness of virtual reality interventions on physiological and psychological outcomes of adults with cardiovascular disease: A systematic review and meta-analysis |
| van der Linde-van den Bora, M., Slond, F., Liesdeka, O., Suykera, W., Weldama, S. | 2022 | The use of virtual reality in patient education related to medical somatic treatment: A scoping review |
| Wang, S., Lim, S., Aloweni, F. | 2022 | Virtual reality interventions and the outcome measures of adult patients in acute care settings undergoing surgical procedures: An integrative review***** |
| Wu, Y., Wang, N., Zhang, H., Sun, X., Wang, Y., Zhang, Y. | 2023 | Effectiveness of Virtual Reality in Symptom Management of Cancer Patients: A Systematic Review and Meta-Analysis |
| Xu, N., Chen, S., Liu, Y., Jing, Y., Gu, P. | 2022 | The Effects of Virtual Reality in Maternal Delivery: Systematic Review and Meta-analysis |
| Yan, X., Yan, Y., Cao, M., Xie, W., O'Connor, S., Lee, J. J., Ho, M. H. | 2023 | Effectiveness of virtual reality distraction interventions to reduce dental anxiety in paediatric patients: A systematic review and meta-analysis |
| Yu, Y., Zhou, X., Zeng, G., Hou, Y. | 2023 | Impact of virtual operating room tours on relieving perioperative anxiety in adult patients: a systematic review |
| Zhang, H., Xu, H., Zhang, Z., Zhang, Q. | 2022 | Efficacy of virtual reality-based interventions for patients with breast cancer symptom and rehabilitation management: a systematic review and meta-analysis |
| *denotes priority review articles, which were used to inform the synthesis of trial-level data | | |

| **Supplementary Table 2.** Trial articles included in our analyses. | | |
| --- | --- | --- |
| **Authors** | **Year** | **Article Title** |
| Aminabadi, N. A., Erfanparast, L., Sohrabi, A., Oskouei, S. G., Naghili, A. | 2012 | The Impact of Virtual Reality Distraction on Pain and Anxiety during Dental Treatment in 4-6 Year-Old Children: a Randomized Controlled Clinical Trial |
| Mohammad, E., Ahmad, M. | 2018 | Virtual reality as a distraction technique for pain and anxiety among patients with breast cancer: A randomized control trial |
| Bekelis, K., Calnan, D., Simmons, N., MacKenzie, T., Kakoulides, G. | 2017 | Effect of an Immersive Preoperative Virtual Reality Experience on Patient Reported Outcomes: A Randomized Controlled Trial |
| Canares, T., Parrish, C., Santos, C., Badawi, A., Stewart, A., Kleinman, K., Psoter, K., McGuire, J., | 2021 | Pediatric Coping During Venipuncture With Virtual Reality: Pilot Randomized Controlled Trial |
| Chan, E., Chung, J., Wong, T., Lien, A., Yang, J. | 2007 | Application of a virtual reality prototype for pain relief of pediatric burn in Taiwan |
| Chan, E., Hovenden, M., Ramage, E., Ling, N., Pham, J., Rahim, A., Lam, C., Liu, L., Foster, S., Sambell, R., Jeyachanthiran, K., | 2019 | Virtual Reality for Pediatric Needle Procedural Pain: Two Randomized Clinical Trials |
| Chan, J., Yeam, C., Kee, H., Tan, C., Sultana, R., Sia, A., Sng, B. | 2020 | The use of pre-operative virtual reality to reduce anxiety in women undergoing gynecological surgeries: A prospective cohort study |
| Clerc, P., Arneja, J., Zwimpfer, C., Behboudi, A., Goldman, R. | 2021 | A Randomized Controlled Trial of Virtual Reality in Awake Minor Pediatric Plastic Surgery Procedures |
| Das, D., Grimmer, K., Sparnon, A., McRae, S., Thomas, B. | 2005 | The efficacy of playing a virtual reality game in modulating pain for children with acute burn injuries: A randomized controlled trial |
| Dehghan, F., Jalali, R., Bashiri, H. | 2019 | The effect of virtual reality technology on preoperative anxiety in children: a Solomon four-group randomized clinical trial |
| Deo, N., Khan, K., Mak, J., Allotey, J., Gonzalez Carreras, F., Fusari, G., Benn, J. | 2021 | Virtual reality for acute pain in outpatient hysteroscopy: a randomised controlled trial |
| Dunn, A., Patterson, J., Biega, C., Grishchenko, A., Luna, J., Stanek, J., Strouse, R. | 2019 | A Novel Clinician-Orchestrated Virtual Reality Platform for Distraction During Pediatric Intravenous Procedures in Children With Hemophilia: Randomized Controlled Trial |
| Eijlers, R., Dierckx, B., Staals, L., Berghmans, J., van der Schroeff, M., Strabbing, E., Wijnen, R., Hillegers, M., Legerstee, J., Utens, E. | 2019 | Virtual reality exposure before elective day care surgery to reduce anxiety and pain in children: A randomised controlled trial |
| Ganry, L., Hersant, B., Sidahmed-Mezi, M., Dhonneur, G., Meningaud, J. | 2018 | Using virtual reality to control preoperative anxiety in ambulatory surgery patients: A pilot study in maxillofacial and plastic surgery |
| Özalp Gerçeker, G., Ayar, D., Özdemir, E., Bektaş, M | 2020 | Effects of virtual reality on pain, fear and anxiety during blood draw in children aged 5–12 years old: A randomised controlled study |
| Gershon, J., Zimand, E., Lemos, R., Rothbaum, B., Hodges, L. | 2004 | Use of Virtual Reality as a Distractor for Painful Procedures in a Patient with Pediatric Cancer: A Case Study |
| Gold, J., Kim, S., Kant, A., Joseph, M., Rizzo, A. | 2006 | Effectiveness of Virtual Reality for Pediatric Pain Distraction during IV Placement |
| Gold, J., Mahrer, N. | 2018 | Is Virtual Reality Ready for Prime Time in the Medical Space? A Randomized Control Trial of Pediatric Virtual Reality for Acute Procedural Pain Management |
| Gold, J., SooHoo, M., Laikin, A., Lane, A., Klein, M. | 2021 | Effect of an Immersive Virtual Reality Intervention on Pain and Anxiety Associated With Peripheral Intravenous Catheter Placement in the Pediatric Setting: A Randomized Clinical Trial |
| Goldman, R., Behboudi, A. | 2021 | Pilot Randomized Controlled Trial of Virtual Reality vs. Standard-of-Care During Pediatric Laceration Repair |
| Gujjar, K., van Wijk, A., Kumar, R., de Jongh, A. | 2019 | Eﬃcacy of virtual reality exposure therapy for the treatment of dental phobia in adults: A randomized controlled trial |
| Gupta, A., Thomas, J. | 2019 | Use of Virtual Reality as a Surrogate for Parental Presence During Anesthetic Induction: A Case Report |
| Haisley, K., Straw, O., Müller, D., Antiporda, M., Zihni, A., Reavis, K., Bradley, D., Dunst, C. | 2020 | Feasibility of implementing a virtual reality program as an adjuvant tool for peri-operative pain control; Results of a randomized controlled trial in minimally invasive foregut surgery |
| Hoffman, H., Doctor, J., Patterson, D., Carrougher, G., Furness, T. | 2000 | Virtual reality as an adjunctive pain control during burn wound care in adolescent patients |
| Hoxhallari, E., Behr, I., Bradshaw, J., Morkos, M., Haan, P., Schaefer, M., Clarkson, J. | 2019 | Virtual Reality Improves the Patient Experience during Wide-Awake Local Anesthesia No Tourniquet Hand Surgery: A Single-Blind, Randomized, Prospective Study |
| Jeffs, D., Dorman, D., Brown, S., Files, A., Graves, T., Kirk, E., Meredith-Neve, S., Sanders, J., White, B., Swearingen, C. | 2014 | Effect of Virtual Reality on Adolescent Pain During Burn Wound Care |
| Jiang, M., Upton, E., Newby, J. | 2020 | A randomised wait-list controlled pilot trial of one-session virtual reality exposure therapy for blood-injection-injury phobias |
| Jung, M., Libaw, J., Ma, K., Whitlock, E., Feiner, J., Sinskey, J. | 2021 | Pediatric Distraction on Induction of Anesthesia with Virtual Reality (PEDI-VR) and Perioperative Anxiolysis: A Randomized Controlled Trial |
| Khadra, C., Ballard, A., Déry, J., Paquin, D., Fortin, J., Perreault, I., Labbe, D., Hoffman, H., Bouchard, S., LeMay, S., | 2018 | Projector-based virtual reality dome environment for procedural pain and anxiety in young children with burn injuries: a pilot study |
| Konstantatos, A., Angliss, M., Costello, V., Cleland, H., Stafrace, S. | 2009 | Predicting the effectiveness of virtual reality relaxation on pain and anxiety when added to PCA morphine in patients having burns dressings changes |
| Koticha, P., Katge, F., Shetty, S., Patil, D. | 2019 | Effectiveness of Virtual Reality Eyeglasses as a Distraction Aid to Reduce Anxiety among 6–10-year-old Children Undergoing Dental Extraction Procedure |
| Lahti, S., Suominen, A., Freeman, R., Lähteenoja, T., Humphris, G. | 2020 | Virtual Reality Relaxation to Decrease Dental Anxiety |
| Lee, H., Bae, W., Park, J., Jung, J., Hwang, S., Kim, D., Kwak, Y. | 2021 | Virtual reality environment using a dome screen for procedural pain in young children during intravenous placement: A pilot randomized controlled trial |
| Liu, K., Ninan, S., Laitman, B., Goldrich, D., Iloreta, A., Londino, A. | 2021 | Virtual Reality as Distraction Analgesia and Anxiolysis for Pediatric Otolaryngology Procedures |
| Marquess, M., Johnston, S., Williams, N., Giordano, C., Leiby, B., Hurwitz, M., Dicker, A., Den, R. | 2017 | A pilot study to determine if the use of a virtual reality education module reduces anxiety and increases comprehension in patients receiving radiation therapy |
| McSherry, T., Atterbury, M., Gartner, S., Helmold, E., Searles, D., Schulman, C. | 2018 | Randomized, Crossover Study of Immersive Virtual Reality to Decrease Opioid Use During Painful Wound Care Procedures in Adults |
| Mosadeghi, S., Reid, M., Martinez, B., Rosen, B., Spiegel, B. | 2016 | Feasibility of an Immersive Virtual Reality Intervention for Hospitalized Patients: An Observational Cohort Study |
| Niharika, P., Reddy, N., Srujana, P., Srikanth, K., Daneswari, V., Geetha, K. | 2018 | Effects of distraction using virtual reality technology on pain perception and anxiety levels in children during pulp therapy of primary molars |
| Noben, L., Goossens, S., Truijens, S., Van Berckel, M., Perquin, C., Slooter, G., Van Rooijen, S. | 2019 | A Virtual Reality Video to Improve Information Provision and Reduce Anxiety Before Cesarean Delivery: Randomized Controlled Trial |
| Nunna, M., Dasaraju, R., Kamatham, R., Mallineni, S., Nuvvula, S. | 2019 | Comparative evaluation of virtual reality distraction and counter-stimulation on dental anxiety and pain perception in children |
| Oyama, H., Kaneda, M., Katsumata, N., Akechi, T., Ohsuga, M. | 2000 | Using the Bedside Wellness System During Chemotherapy Decreases Fatigue and Emesis in Cancer Patients |
| Ran, L., Zhao, N., Fan, L., Zhou, P., Zhang, C., Yu, C. | 2021 | Application of virtual reality on non-drug behavioral management of short-term dental procedure in children |
| Robertson, A., Khan, R., Fick, D., Robertson, W., Gunaratne, D., Yapa, S., Bowden, V., Hoffman, H., Rajan, R. | 2017 | The effect of virtual reality in reducing preoperative anxiety in patients prior to arthroscopic knee surgery: A randomised controlled trial |
| Ryu, J., Park, S., Park, J., Kim, J., Yoo, H., Kim, T., Hong, J., Han, S. | 2017 | Randomized clinical trial of immersive virtual reality tour of the operating theatre in children before anaesthesia |
| Ryu, J., Park, J., Nahm, F., Jeon, Y., Oh, A., Lee, H., Kim, J., Han, S. | 2018 | The Effect of Gamiﬁcation through a Virtual Reality on Preoperative Anxiety in Pediatric Patients Undergoing General Anesthesia: A Prospective, Randomized, and Controlled Trial |
| Ryu, J., Oh, A., Yoo, H., Kim, J., Park, J., Han, S. | 2019 | The effect of an immersive virtual reality tour of the operating theater on emergence delirium in children undergoing general anesthesia: A randomized controlled trial |
| Sahin, G., Basak, T. | 2020 | The Effects of Intraoperative Progressive Muscle Relaxation and Virtual Reality Application on Anxiety, Vital Signs, and Satisfaction: A Randomized Controlled Trial |
| Schlechter, A., Whitaker, W., Iyer, S., Gabriele, G., Wilkinson, M. | 2021 | Virtual reality distraction during pediatric intravenous line placement in the emergency department: A prospective randomized comparison study |
| Schneider, S., Workman, M. | 1999 | Effects of Virtual Reality on Symptom Distress in Children Receiving Chemotherapy |
| Schneider, S., Ellis, M., Coombs, W., Shonkwiler, E., Folsom, L. | 2003 | Virtual Reality Intervention for Older Women with Breast Cancer |
| Schneider, S., Prince-Paul, M., Allen, M., Silverman, P., Talaba, D. | 2004 | Virtual Reality as a Distraction Intervention for Women Receiving Chemotherapy |
| Schneider, S., Hood, L. | 2007 | Virtual Reality: A Distraction Intervention for Chemotherapy |
| Sweta, V., Abhinav, R., Ramesh, A. | 2019 | Role of Virtual Reality in Pain Perception of Patients Following the Administration of Local Anesthesia |
| Tanja-Dijkstra, K., Pahl, S., White, M., Andrade, J., Qian, C., Bruce, M., May, J., Moles, D. | 2014 | Improving Dental Experiences by Using Virtual Reality Distraction: A Simulation Study |
| van Twillert, B., Bremer, M., Faber, A. | 2007 | Computer-Generated Virtual Reality to Control Pain and Anxiety in Pediatric and Adult Burn Patients During Wound Dressing Changes |
| Wolitzky, K., Fivush, R., Zimand, E., Hodges, L., Rothbaum, B. | 2005 | Effectiveness of virtual reality distraction during a painful medical procedure in pediatric oncology patients |
| Wong, C., Li, C., Chan, C., Choi, K., Chen, J., Yeung, M., Chan, O. | 2021 | Virtual Reality Intervention Targeting Pain and Anxiety Among Pediatric Cancer Patients Undergoing Peripheral Intravenous Cannulation: A Randomized Controlled Trial |
| Xiang, H., Shen, J., Wheeler, K., Patterson, J., Lever, K., Armstrong, M., Shi, J., Thakkar, R., Groner, J., Noffsinger, D., Giles, S. | 2021 | Efficacy of Smartphone Active and Passive Virtual Reality Distraction vs Standard Care on Burn Pain Among Pediatric Patients: A Randomized Clinical Trial |
| Yang, J., Ryu, J., Nam, E., Lee, H., Lee, J. | 2019 | Effects of Preoperative Virtual Reality Magnetic Resonance Imaging on Preoperative Anxiety in Patients Undergoing Arthroscopic Knee Surgery: A Randomized Controlled Study |
